# Alcohol binge drinking induces downregulation of blood-brain barrier proteins in the rat frontal cortex -but not in the hippocampus- that is not prevented by OEA pretreatment

**DOI:** 10.3389/adar.2023.11091

**Published:** 2023-02-24

**Authors:** Alicia Rodríguez-González, Marta Moya, Fernando Rodríguez de Fonseca, Raquel Gómez de Heras, Laura Orio

**Affiliations:** ^1^ Department of Psychobiology and Behavioral Sciences Methods, Faculty of Psychology, Complutense University of Madrid, Madrid, Spain; ^2^ RIAPAd: Research Network in Primary Care in Addictions (Red de Investigación en Atención Primaria en Adicciones), Madrid, Spain; ^3^ Instituto de Investigación Sanitaria Hospital Universitario 12 de Octubre (imas12), Madrid, Spain

**Keywords:** alcohol, neuroinflammation, blood-brain-barrier, BBB, oleoylethanolamide, tight junctions, occludin, laminin

## Abstract

Alcohol binge drinking promotes neuroinflammation which could be partially mediated by the passage of ABD-induced peripheral inflammatory molecules to the brain parenchyma through the blood-brain barrier. The BBB is sealed by tight junction proteins, which regulate the access of substances to the brain. Whether ABD alters the BBB or not remains controversial. Here, we measured the expression of BBB proteins in frontal cortex and hippocampus after an ABD procedure that was previously shown to induce neuroinflammation in the FC, and checked neuroinflammation in the hippocampus. Oleoylethanolamide is known to inhibit ABD-induced neuroinflammation in rat FC but the mechanisms of action are not clear: whereas OEA protects against alcohol-induced breakdown of the TJ proteins in the gut barrier reducing peripheral inflammation, its effect in the TJ of the BBB remains unknown. Here, we studied whether OEA (5 mg/kg, before each gavage) prevented alcohol-induced BBB dysfunction by measuring the expression of zona-occludens, occludin, and laminin in FC and hippocampus. ABD animals showed reduced laminin and occludin levels in the FC, indicative of BBB dysfunction, which is concordant with previous findings showing ABD-induced neuroinflammation in this brain region. OEA did not prevent ABD-induced changes in the BBB proteins in the FC, suggesting that the OEA main mechanism of action to inhibit neuroinflammation in this brain region is not related to prevention of TJ proteins alteration in the BBB. In the hippocampus, this ABD protocol did not alter BBB protein levels and no markers of neuroinflammation were found elevated.

## Introduction

Alcohol binge drinking (ABD) is defined as the consumption of more than four or five drinks in about 2 h, resulting in blood alcohol levels (BAL) ≥80 mg/dL (1). 38 millions of US adults drink alcohol in this pattern, resulting in high rates of mortality (2).

ABD induces alterations in the morphology of frontal cortex (FC) and hippocampus, which have been correlated with cognitive dysfunctions, i.e., impaired memory (3). ABD promotes neuroinflammation by the activation of the innate immunity Toll-like receptor 4 (TLR4) in the brain, inducing the release of pro-inflammatory molecules, such as tumour necrosis factor-α (TNF-α) and interleukin-1β (IL-1β), which could induce neuronal damage if maintained in the long-term (3, 4). Alcohol consumption also promotes the release of pro-inflammatory molecules from the gut and liver to the blood, which could cross the blood-brain barrier (BBB) reaching the brain and exacerbating the neuroinflammation (5, 6). Up to date, the effects of ABD in the BBB are scarce and controversial, some reports indicate decreased BBB protein levels and increased BBB permeability (7, 8), while others show no changes in BBB permeability after ABD (9).

The BBB is one of the most selective biological barriers, which regulates the passage of blood oxygen and nutrients to the brain parenchyma, protecting the brain from toxins and pathogens (10). It is formed by specialized endothelial cells sealed by tight-junctions (TJ), composed by the transmembrane proteins occludin and claudins, and the cytoplasmic protein *zona occludens* (ZO-1) bound to the cytoskeleton. These endothelial cells are surrounded by the basal lamina, an extracellular matrix composed by laminin and other molecules (11, 12). TJ proteins constitute the first and most important barrier sealing the BBB preventing the passage of molecules through the paracellular *via* by diffusion (12), and TJ breakdown is linked to BBB dysfunction in different conditions (10).

We have previously shown in our laboratory that the 4-day binge paradigm promotes neuroinflammation in the FC: i.e., increased levels of TLR4, TNF-α, IL-1β (13). However, whether this 4-day ABD protocol induces neuroinflammation in the hippocampus remains controversial: Zhao et al. reported inflammation in the hippocampus (14), while Marshall et al. did not find increased levels of inflammatory parameters in this brain region (15). Despite much progress being made in this area, the specific contribution of peripheral versus central immune response in ETOH-induced neuroimmune activation and BBB integrity remains unconclusive. Peripheral cytokines and/or the direct effect of ethanol on the brain TLR4 signalling pathway induce a proinflammatory environment related to neuroinflammation. An injured BBB amplify the brain damage (5).

Our laboratory has also shown that the pharmacological pre-treatment with the endogenous biolipid oleoylethanolamide (OEA) during ABD reduced neuroinflammation and has neuroprotective effects in the FC of rats (13). As well as it happens with the effects of alcohol inducing inflammation, both central and peripheral mechanisms have been suggested for OEA neuroprotective actions (16). Indeed, the diffusion of OEA into the brain has been previously documented (17) and recently, a peripheral mechanism of OEA regulating the function of the intestinal barrier has been described by our laboratory, since OEA pre-treatment reduced ABD-induced leaky gut in the colon and peripheral inflammation (18). Whereas OEA has been shown to prevent the downregulation of TJ proteins induced by ABD in the gut, limiting partially the entry of proinflammatory components to the systemic circulation, its possible action in the modulation of TJ proteins of the BBB has not been explored yet. A protective action of OEA in the BBB may prevent the entry of proinflammatory molecules through an impaired barrier in ABD conditions from the periphery to the brain, with consequences in neuroinflammation. Alternatively, a direct effect of OEA reducing neuroinflammation may preserve alterations in the BBB which presumably enhances the neuroinflammatory process.

The aim of this study was to check the integrity of some proteins conforming the BBB after ABD in the FC, an area in which neuroinflammation was extensively documented after a 4-day ABD protocol, and in the hippocampus, in which we explored the potential of ABD to induce neuroinflammation. Since OEA has previously shown protective actions in the ABD-induced TJ disruption in the gut barrier, we tested the possible protective effect of OEA in the integrity of other biological barrier, the BBB, if altered by alcohol. Discussion about the implications for the prevention of neuroinflammation is provided.

## Materials and methods

### Animals

Fifty adult male Wistar rats (Envigo, Barcelona, Spain) of 7–8 weeks old and 260–330 g were used, housed in groups of three to four rats per cage on a reverse 12 h light/dark cycle under standard temperature and humidity at the SPF Animal Care Facility of the Complutense University of Madrid. Animals were allowed free access to tap water and standard food (A04 SAFE, Scientific Animal Food and Engineering, Augy, France) and maintained 10 days under constant conditions before the experiments, with daily surveillance. All studies were designed in compliance with the ARRIVE guidelines (19) and adhered to the guidelines of the Animal Welfare Committee of the Complutense University of Madrid (reference: PROEX 420/15) according to European legislation (2010/63/UE).

### Drugs

Alcohol (30%, v/v) or vehicle (tap water) was intragastrically administered at a maximum of three times/day using specific canulae (16G needle, Fisher Scientific, Waltham, MA, United States) during four consecutive days, following a standard paradigm of ABD more deeply explained in (13, 16). The doses were tritated by observing the behavioural signs related to alcohol intoxication and by BAL. The average dose of alcohol per rat was 8.12 g/kg/day in this experiment. Number of rats/group was initially higher in the ethanol groups, to control for possible binge-induced mortality. This protocol of ABD led to a sedation/ataxia behaviour, relatively constant intoxicating BALs (average per group/day: ethanol 198.01 ± 14.83 mg/dL; OEA + ethanol 230139 ± 14.36 mg/dL) and induced around 7% of mortality, similarly to other studies (13, 16). OEA, synthesized as in (20), was dissolved in 5% Tween 80 in saline and administered at 5 mg/kg, i.p., or the vehicle (5% Tween 80 in saline), 10 min before the alcohol binges, with the exception of the loading initial dose that was 10 mg/kg. This protocol of OEA administration has demonstrated to reduce ABD-induced peripheral inflammation and neuroinflammation (13, 16).

### BAL determination and tissue collection

Blood samples were collected from the rat tail 2 h before the second alcohol administration every experimental day (at 3 p.m.). 20 μL of blood from each rat were used to measure BAL by enzymatic reaction using electrochemical detection with AM1 Alcohol Analyzer (Analox Instruments, London, United Kingdom).

Brain tissue samples from frontal cortex and hippocampus were collected 3 h after the last binge of alcohol following a lethal dose of sodium pentobarbital (300 mg/kg, i.p., Dolethal^®^, Spain) and stored at −80°C until assayed.

### Western blot

Samples were homogenized by sonication in 250 (hippocampus) or 300 (frontal cortex) µL of PBS (pH = 7.4) containing a protease inhibitor cocktail (Complete, Roche^®^, Madrid, Spain), followed by a centrifugation at 12,000 g for 10 min at 4°C. Homogenates were adjusted to the same protein levels and were mixed with Laemmli sample buffer (BioRad^®^, Alcobendas, Madrid, Spain) containing β-mercaptoethanol (50 μL/mL of Laemmli). 1 mg/mL of protein was loaded into each electrophoresis gel.

**FIGURE 1:**
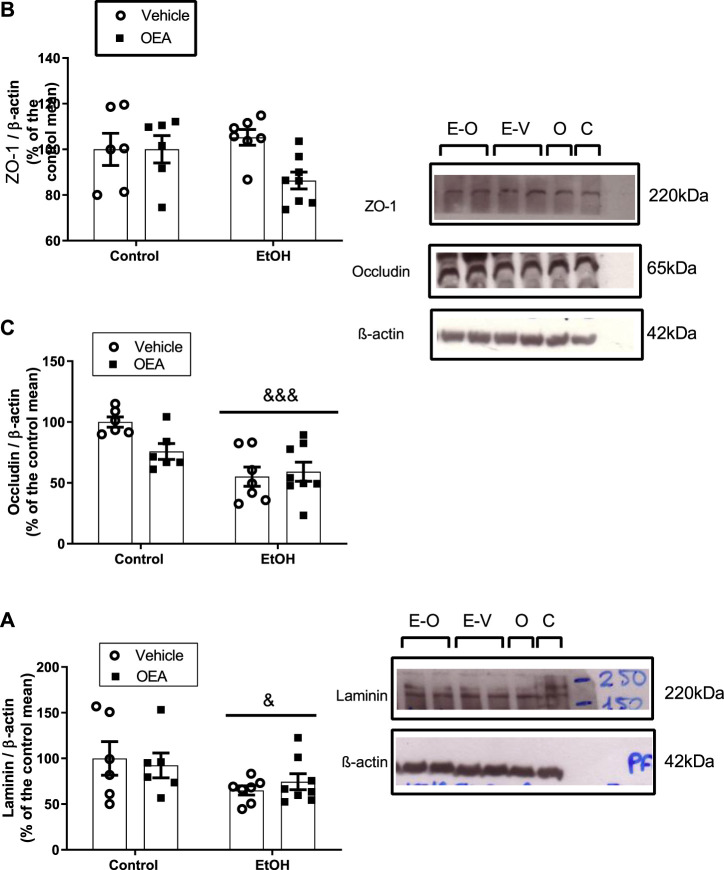
Effects of alcohol binge drinking in the BBB proteins in the rat frontal cortex. Protein levels of ZO-1 **(A)** and occludin **(B)** and laminin **(C)**. ZO-1 and occluding were obtained from the same blot by cutting specifics parts and incubate them with specific antibodies. Images show the aspect of the proteins in the transferred membranes. Data were normalized by β-actin. Data in graph represent the mean ± SEM (E-O: EtOH + OEA *n* = 8; E-V: EtOH + Vehicle *n* = 7; O: Control + OEA *n* = 6; C: Control + Vehicle *n* = 6). Alcohol binge drinking alters the expression of laminin and occludin, with no effect in ZO-1. Pre-treatment with OEA did not prevent alterations in the expression of BBB proteins induced by alcohol. Data were normalized with β-actin. Statistical analysis: two-way analysis of variance (ANOVA), followed by Bonferroni’s *post-hoc* test. Overall effect of alcohol: ^&^ *p* < 0.05; ^&&&^ *p* < 0.001.

**FIGURE 2:**
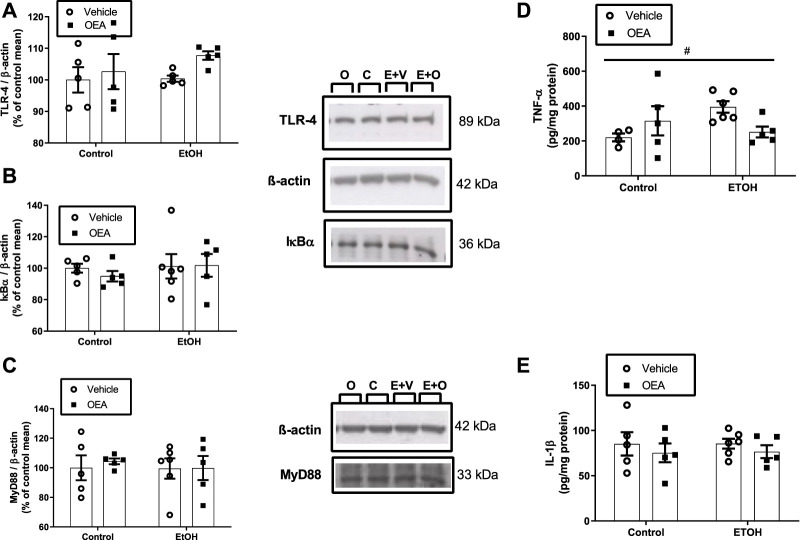
Effects of alcohol binge drinking in neuroinflammatory parameter in the hippocampus. Levels of TLR4 **(A)**, IκBα **(B)** and MyD88 **(C)** were measured by western blot. Protein levels of TLR4 and IκB-α were extracted from the same gel and MyD88 from a different gel. Data were normalized by β-actin. C: Control + Vehicle *n* = 5; O: Control + OEA *n* = 5; E+V: EtOH + Veh *n* = 5-6; E+O: EtOH + OEA *n* = 5). Levels of TNF-α **(D)** and IL-1β **(E)** were measured by ELISA. Data are shown as the mean ± S.E.M. 2-way ANOVA: interaction effect between variables ^#^ *p* < 0.05.

Proteins were blotted onto a nitrocellulose membrane (Amersham Ibérica^®^, Madrid, Spain) in a semi-dry transfer system (Bio-Rad Alcobendas, Madrid, Spain), and were incubated with specific primary and secondary antibodies (Laminin, dilution 1:1,000, Merck KGaA^®^, Darmstadt, Germany; ZO-1, 1:750 in 1.5% BSA, Santa Cruz Biotechnology^®^, CA, United States; occludin, 1:1,000, Invitrogen, Grand Island, NY, United States; β-Actin, 1:15,000, Merck KGaA^®^, Darmstadt, Germany; TLR4, 1:500 in 1% BSA, Santa Cruz Biotechnology^®^, CA, United States; MyD88, 1:750 in 5% BSA, Abcam^®^, Cambridge, United Kingdom; iκBα, 1:1,000 in 5% BSA, Santa Cruz Biotechnology^®^, CA, United States). Selected proteins were revealed using ECL™ kit (Amersham Ibérica, Madrid, Spain). β-Actin (42 kDa) was used as loading control. The same β-actin lane was used as loading control for: a) ZO-1 and occluding; b) TLR4 and IκBα, as they were analysed in the same western blot gels. Membranes were cut at specific levels to allow determination of the different proteins, which were revealed manually, so different exposure times were used to ensure the optimal visualization of the band intensities. The band intensity of the autoradiographs was quantified by densitometry analysis with ImageJ (NIH ImageJ^®^ software, National Biosciences, Lincoln, NE, United States). Raw densitometric data were expressed as optical density (OD) and transformed as fold change of the control mean. Representative blots are shown in Figures 1, 2 including *n* = 1–2 immunoblots images for each experimental group in an uncropped manner. Quantification results (densitometries) are shown in figures using scattered data to improve clarity, showing the mean ± SEM of densitometries.

### Proinflammatory cytokine levels in hippocampus

Protein levels of IL-1β and TNF-α were measured in the hippocampus using commercially available enzyme-linked immunosorbent assay (ELISA) kits (RayBiotech^®^, Norcross, Georgia) following the manufacturer’s instructions. Hippocampal samples were prepared as explained for Western blot analysis.

### Statistics

Data in text and figures are expressed as mean ± SEM. Normality was tested by Kolmogorov-Smirnov test and variance homogeneity by the Bartlett’s test. Data were analysed by two-way analysis of variance (ANOVA) with the factors [alcohol/water] versus [OEA/vehicle], with a Bonferroni *post-hoc* test used when appropriate. A *p*-value ≤0.05 was considered as statistically significant. Data were analysed using GraphPad Prism 6 for Windows (GraphPad Software, Inc., La Jolla, CA, United States).

## Results

### Effects of ABD and OEA pre-treatment in BBB tight junction proteins and laminin in frontal cortex

ABD did not promote changes in the protein level of ZO-1 (Figure 1A, F_(1,23)_ = 0.3721, *p* > 0.05, n.s.) in the rat FC, but induced downregulation of the TJ occludin and a decrease in the protein levels of laminin, a component of the basal lamina (Figures 1B, C: 2-way ANOVA, overall alcohol effect F_(1,23)_ = 18.34, *p* < 0.001; F_(1,23)_ = 5.092, *p* < 0.05, respectively).

Regarding the OEA pre-treatment during ABD, OEA did not alter ABD-induced changes in protein levels of occludin or laminin (Figures 1B, C; 2-way ANOVA, no OEA effect: F_(1,23)_ = 1.982, *p* > 0.05, n.s.; F_(1,23)_ = 0.006499, *p* > 0.05, n.s., respectively; and no interaction: F_(1,23)_ = 3.872, *p* > 0.05, n.s.; F_(1,23)_ = 0.5325, *p* > 0.05, n.s.). Data represents the mean ± SEM Control + Vehicle (C) *n* = 6; Control + OEA (O) *n* = 6; EtOH + Vehicle (E + V) *n* = 7; EtOH + OEA (E + O) *n* = 8.

### Effects of ABD and OEA pre-treatment in the BBB proteins of the rat hippocampus

Unlike the FC, no effect of ABD was found in the laminin, occludin and ZO-1 protein levels in the rat hippocampus (Table 1; 2-way ANOVA, F_(1,17)_ = 0.09565, *p* > 0.05, n.s.; F_(1,16)_ = 0.1941, *p* > 0.05, n.s.; F_(1,16)_ = 0.06990, *p* > 0.05, n.s., respectively).

**TABLE 1:** Effects of alcohol binge drinking in the BBB proteins in the rat hippocampus.

	Laminin	Occludin	ZO-1
Control	100.00 ± 7.42	100,00 ± 5.13	100.00 ± 8.68
OEA + control	85,31 ± 10.74	104.35 ± 9.07	119.63 ± 13.76
EtOH + vehicle	1,001.56 ± 15.96	106.72 ± 7.87	105.32 ± 13.13
OEA + EtOH	94.74 ± 29.05	103.63 ± 8,66	106.83 ± 12.24

Protein levels of laminin, occludin and ZO-1. Data represent the mean ± SEM (Control + Vehicle *n* = 5; Control + OEA *n* = 4–5; EtOH + Vehicle *n* = 6; EtOH + OEA *n* = 5). Data were normalized with β-actin. ABD did not affect BBB protein levels in the rat hippocampus. Statistical analysis: two-way analysis of variance (ANOVA), followed by Bonferroni’s *post-hoc* test; *p* > 0.05, n.s.

Pre-treatment with OEA did not affect those proteins neither in the alcohol nor in the control groups (Table 1; 2-way ANOVA, no OEA effect: F_(1,17)_ = 0.3670, *p* > 0.05, n.s.; F_(1,16)_ = 0.01524, *p* > 0.05, n.s.; F_(1,16)_ = 0.6908, *p* > 0.05, n.s.; respectively; and no interaction: F_(1,17)_ = 0.04906, *p* > 0.05, n.s.; F_(1,16)_ = 0.2538, *p* > 0.05, n.s.; F_(1,16)_ = 0.5117, *p* > 0.05, n.s.; respectively). Data represents the mean ± SEM Control + Vehicle *n* = 5; Control + OEA *n* = 4–5; EtOH + Veh *n* = 6; EtOH + OEA *n* = 5.

### Neuroinflammation in the hippocampus after ABD and pre-treatment with OEA

The previous results presented in this article indicate that ABD alters the BBB protein levels in the rat FC, an area that has previously shown to have upregulated levels of neuroinflammatory markers after ABD. Since ABD did not affect BBB protein levels in the hippocampus, we studied the expression of key neuroinflammatory parameters in this brain region.

Thus, to explore whether ABD induces hippocampal neuroinflammation we evaluated the influence of ABD on the levels of the receptor TLR4 and the expression of the myeloid differentiation factor 88 (MyD88), an adaptor molecule of a TLR4 intracellular signaling pathway that mediates the translocation of the Nuclear Factor-κB (NF-κB) to the nucleus, activating the transcription of proinflammatory molecules, such as TNF-α and IL-1β (21), which were also measured. We also checked the protein levels of the NF-κB inhibitory protein I kappa B-α (IκB-α).

No effects of ABD were found on the protein levels of TLR4, MyD88, IκB-α (Figures 2A–C; 2-way ANOVA F_(1,16)_ = 0.6047, *p* > 0.05, n.s.; F_(1,17)_ = 0.1318, *p* > 0.05, n.s.; F_(1,17)_ = 0.4481, *p* > 0.05, n.s., respectively) and no effect of OEA pretreatment was found in these proteins neither in the alcohol nor in the control groups (Figures 2A–C; 2-way ANOVA no OEA effect: F_(1,16)_ = 1.982, *p* > 0.05, n.s.; F_(1,17)_ = 0.1151, *p* > 0.05, n.s.; F_(1,17)_ = 0.1385, *p* > 0.05, n.s.; and no interaction: F_(1,16)_ = 0.4436, *p* > 0.05, n.s.; F_(1,17)_ = 0.08481, *p* > 0.05, n.s.; F_(1,17)_ = 0.2220, *p* > 0.05, n.s. for in TLR4, MyD88 e IκBα, respectively).

Regarding proinflammatory cytokines, TNF-α levels (Figure 2D) in the alcohol group were upregulated 79% over controls (*p* < 0.005, *t*-test) but 2-way ANOVA revealed no main effect of alcohol for TNF-α or IL-1β (Figures 2D, E; F_(1,16)_ = 1.1214, *p* > 0.05, n.s.; F_(1,17)_ = 0.006256, *p* > 0.05, n.s., respectively). An interaction between factors/F_(1,16)_ = 5.604, *p* < 0.05) was found for TNF-α, with no significant *post-hoc* test; *p* > 0.05, n.s.). Pre-treatment with OEA did not affect protein levels of TNF-α or IL-1β neither in the alcohol nor in the control groups (Figures 2D, E; 2-way ANOVA F_(1,16)_ = 0.2340, *p* > 0.05, n.s.; F_(1,17)_ = 1.043, *p* > 0.05, n.s., respectively. Data represents the mean ± SEM Control + Vehicle (C) *n* = 5; Control + OEA (O) *n* = 5; EtOH + Veh (E + V) *n* = 5–6; EtOH + OEA (E + O) *n* = 5.

## Discussion

TJ proteins have an essential role in the formation and maintenance of the BBB function, conferring structural integrity and low permeability (22). Chronic alcohol alters the BBB by decreasing TJ proteins, which increases the barrier permeability and neuroinflammation (23). However, studies assessing the effect of ABD in the BBB are still scarce. Here, we found regional differences in the impact that ABD has in some BBB protein levels. ABD alters the basal lamina component laminin and the TJ protein occluding in the rat FC, with no effect in ZO-1 protein levels. The absence of effect in the levels of ZO-1 found in this study did not discard other alterations in this protein, since it has been showed that IL-1β induces BBB dysfunction by phosphorylation of ZO-1 with no changes in the expression levels of the protein (23).

Whereas ABD induced the downregulation of laminin and occluding in the rat FC, no effect was found in the hippocampus. The degradation of BBB proteins after chronic alcohol consumption has been previously associated with BBB leakiness leading to apoptosis and neuroinflammation *via* activation of caspase-1 and IL-1β release (23). Consistently, in the ABD model used in this study in which we observe degradation of some BBB proteins we previously reported a strong neuroinflammation in the FC by upregulation of TLR4, MyD88, IκB-α, TNF-α and IL-1β (13), key proinflammatory parameters. As mentioned before, here we show also no significant changes in the BBB proteins in the hippocampus after ABD and no signs of neuroinflammation were found in this brain region. These results suggest a link between BBB protein degradation and the presence of neuroinflammation after alcohol abuse, although we cannot determine the direction (causality or consequence) of such association.

It is hypothesized that the strong peripheral inflammation induced by ABD may cause a damage in some components of the BBB, allowing the entry of proinflammatory components and immune cell infiltration from the systemic circulation to the brain, which may contribute to ABD-induced neuroinflammation observed in FC (16). Alternatively, a direct action of alcohol in the brain may induce neuroinflammation, which could contribute to the structural and functional alterations of the BBB (5). Both phenomena may even occur simultaneously. In this study, ABD did not alter BBB protein levels in the hippocampus and, in accordance with the previous mentioned hypotheses, there was not a clear neuroinflammatory signature in this brain region. Regarding the first hypothesis, the reasons explaining why peripheral inflammation may weaken the BBB in the FC but not in the hippocampus are unclear at present, although it has been demonstrated than the FC is more vulnerable to the oxidative stress and neuronal death induced by chronic alcohol than the hippocampus (24).This could account also for the second hypothesis, the local action of alcohol in the brain, since regional specific actions of alcohol have been described previously. For example, alcohol intermittent exposure in adolescents leads to impairments on frontocortical tasks whereas hippocampal functional deficits begin to emerge, with only sparing on most hippocampal-dependent tasks (25). One possible explanation for these regional differences would be that the hippocampus had additional neuroprotective mechanisms than the FC. Our results are then in agreement with previous reports showing differential sensitivity of FC and hippocamps to alcohol (24) and suggest that the FC could be more vulnerable than the hippocampus to the toxic effects of alcohol after bingeing, in terms of both BBB disruption and neuroinflammation.

Our results showing no effect of ABD in the hippocampal BBB agree with a report showing a functionally intact BBB in the rat hippocampus using the same 4-day ABD protocol (9). This study also reported absence of hippocampal inflammation, which agrees with the lack of ABD-induced inflammation in the rat hippocampus observed in the present study. Additionally, other authors have also reported lack of inflammation in the hippocampus using the same 4-day ABD protocol (15, 26) [but also see (14)]. It is to note that some authors reported increased MyD88 levels in the hippocampus of mice exposed to other protocols of ABD (7) and some functional studies also showed evidence of BBB disruption using other animal models of ABD (7, 8). In the present study we observed only a TNF-α increment after ABD in this brain region, suggesting an initiation or a weak neuroinflammatory signature in the hippocampus, which may be more resistant than the FC to the actions of ABD, as mentioned before in this discussion and pointed by others (24, 25).

Although the hippocampus was shown to be as rich in microvessels as the FC, pathological processes may affect differently several components of the BBB in each brain region. For example, in mouse models of both aging and Alzheimer’s disease there is a decrease in vessel densities in the hippocampus, while in the cortex loss of microvascularization was a consequence of the interaction of both pathologies. Although aging induced capillary loss in all brain regions, the severity of this process was higher in the grey matter than in the white matter, and reduction of capillary branch points affected the cortex and the white matter but not the hippocampus (27). Indeed, much of the structural and functional differences in the BBB among different brain regions are still fully unknown.

To our very best knowledge, this is the first study reporting an ABD-induced reduction of BBB protein levels in the FC. Due to the essential role that TJ proteins and basal lamina have in the integrity of the barrier, this reduction could be suggesting that ABD may promote an increase in the permeability of the BBB. However, functional studies using dye-penetrating techniques are required to fully evaluate alterations in the BBB permeability.

Another result of this study is that the pretreatment with the acylethanolamide OEA was not able to prevent ABD-induced changes in BBB protein expression in the FC. This result was unexpected since previous studies in our laboratory showed the potential of OEA in preventing ABD-induced neuroinflammation in FC using the same animal model (13). OEA prevented also the neuroinflammation induced in this brain region by lipopolysaccharide injection (28).

It has been suggested that the effects of alcohol in the disruption of the BBB could be associated to the neuroimmune activation, playing an important role the alcohol-induced oxidative stress and elevated levels of proinflammatory mediators such as cytokines. Specifically, sustained matrix metalloproteinases (MMP) 3/9 activation during oxidative stress causes TJ degradation and the decrease in the vascular endothelial growth factor receptor 2 (VEGFR-2) leading to neuroinflammation *via* the activation of caspase-1 and interleukin-1beta (IL-1β) release (23, 29). It is well known than alcohol abuse induces a strong peripheral inflammation, oxidative stress and neuroinflammation. Both the peripheral inflammation and the neuroinflammation could influence the integrity of the BBB, and it has been reported that astrocytes and oligodendrocytes may have a biphasic effect in the BBB, acting at both sides of the barrier (30). An altered BBB after alcohol abuse may favoured the infiltration of immune cells and proinflammatory molecules to the brain inducing an exacerbation of the neuroinflammation. It is not clear the direction of the evens since in some pathologies (i.e., traumatic brain injury) BBB disruption precedes neuroinflammation and in others (i.e., septic encephalopathy) impairment of BBB integrity was shown to occur in parallel with neuroinflammation.

The neuroprotectant actions of OEA could be mediated by a direct action within the brain and/or related with a potent anti-inflammatory activity peripherally. Indeed, OEA pretreatment was shown to prevent ABD-induced peripheral inflammation and neuroinflammation in FC (13). It is very possible that OEA uses different mechanisms of action to exert its potent anti-inflammatory actions. On one hand, OEA may act directly within the brain, since some studies report that OEA crosses the BBB (17), although this is still controversial (31). On the other hand, OEA was able to partially block the entry of proinflammatory components to the systemic circulation by protecting against ABD-induced leaky gut, reducing peripheral oxidative stress and inflammation (18), which influences neuroinflammation. It is known that OEA binds the antiinflammatory peroxisome proliferator-activated receptor-alpha (PPAR-α) (20), which is expressed in peripheral tissues and within the brain. OEA could also exert its protective effects *via* direct activation of the area postrema (32) or vagal sensory fibres (33), which are the mechanisms through which OEA exerts its satiety effects. In our study, the protective effects of OEA in ABD conditions appears to not be related to structural changes in the TJ proteins of the FC.I It is to note that the disregulation of the BBB function is a very complex process which includes not only an increase in the paracellular permeability, but also transcytosis and endocytosis mechanisms and caspase-mediated hypermeability (30), which have not been explored. Whether OEA is able to alter the transcellular mechanisms in the BBB deserves further investigation. Clearly more studies are needed to ascertain the contribution of peripheral or central inflammation to the disruption of BBB proteins and the specific targets of OEA.

In conclusion, the FC, a targeted area where alcohol induces neuroinflammation, showed alterations in the expression of BBB protein levels after ethanol binges. BBB protein levels were not affected in the hippocampus. As opposed to OEA protective actions in TJ proteins of the gut barrier altered by alcohol (18), no effect of OEA was found preventing alcohol-induced TJ and laminin protein level alterations in FC.

## Data Availability

The original contributions presented in the study are included in the article, further inquiries can be directed to the corresponding author.
